# Microbial community dynamics in the mesophilic and thermophilic phases of textile waste composting identified through next-generation sequencing

**DOI:** 10.1038/s41598-021-03191-1

**Published:** 2021-12-08

**Authors:** Saloua Biyada, Mohammed Merzouki, Taisija Dėmčėnko, Dovilė Vasiliauskienė, Rūta Ivanec-Goranina, Jaunius Urbonavičius, Eglė Marčiulaitienė, Saulius Vasarevičius, Mohamed Benlemlih

**Affiliations:** 1 Laboratory of Biotechnology, Environment, Agrifood, and Health, Faculty of Sciences Dhar El Mahraz, Sidi Mohamed Ben Abdellah University, Atlas, BP: 1796, 30 000 Fez, Morocco; 2 Department of Chemistry and Bioengineering, Vilnius Gediminas Technical University, 10223 Vilnius, Lithuania; 3 Department of Environmental Protection and Water Engineering, Vilnius Gediminas Technical University, 10223 Vilnius, Lithuania

**Keywords:** Biotechnology, Microbiology, Molecular biology, Environmental sciences

## Abstract

Composting is a promising source of mesophilic and thermophilic microorganisms directly involved in the decay of organic matter. However, there is a paucity of information related to bacterial and fungal diversity in compost and their enzymatic activities during the composting process. In this work, bacterial and fungal diversity during the mesophilic and thermophilic phases of textile waste composting was investigated as a way to explain the physical–chemical results obtained during the composting process. This was accomplished using a next-generation sequencing approach that targets either the 16S rRNA or ITS genomic regions of bacteria and fungi, respectively. It was observed that Proteobacteria, Bacteroidetes, and Actinobacteria were the dominant bacterial phyla present at the mesophilic phase but not at the thermophilic one. Composting textile waste exhibits a sustained thermophilic profile (above 55 °C) that usually precludes fungal activity. Nonetheless, the presence of fungi at the thermophilic phase was observed. Rozellomycota, Basidiomycota, and Ascomycota were the most dominant phyla during both composting phases. Such thermophilic fungi with great ability to decay organic matter could be isolated as pure cultures and used for the bioaugmentation of textile waste composting to achieve an advanced maturity level of textile waste compost.

## Introduction

Composting is a self-heating treatment through which the microbial metabolism raises the temperature above 50 °C, followed by a gradual cooling towards the end of process^1^. It is a highly dynamic process that consists of three phases: mesophilic, thermophilic, and maturation^2^. Alternating mesophilic and thermophilic microbial consortia perform the bio-decomposition of organic matter according to distinct requirements and tolerances that are compatible with the continuously changing environment^3^. Microbial metabolic processes play a significant role in decaying recalcitrant carbon- and nitrogen-containing molecules such as cellulose, lignocellulose, and proteins present in the feedstock, resulting in a product that can be used as a fertilizer for plants or as a soil conditioner^4^.

In Morocco, the textile industries generate around 4 tons of solid waste annually. This waste is disposed of into the environment without any form of treatment, and thus represents a significant threat to the functioning of many ecosystems^5^. This waste is a fountain of organic matter, which could be recovered biologically through composting into bio-fertilizer, thus providing the nutrients necessary for plant growth.

Composting is considered a promising source of new mesophilic and thermophilic bacteria and fungi, especially those involved in the degradation of biomass^1^. In line with their outstanding role in composting, microorganisms have aroused the interest of researchers^6^. Despite the number of studies concerning the identification of mesophilic and thermophilic microorganisms during composting, a deeper understanding of specific taxonomic and functional groups is still required^2,3^. Several groups of microorganisms are related to the composting process, with bacteria and fungi being the most prominent^6^. Bacteria are considered the most dominant species during the transformation processes due to their higher thermal tolerance^7^. However, fungi play a significant role in composting by releasing the enzymes (cellulases, phosphatases, etc.) that destroy recalcitrant molecules, which are not easily decomposed by other microorganisms^6^. In addition, they can tolerate change even under the harshest environmental conditions. Indeed, during composting, fungal diversity is influenced by changes in physical–chemical parameters such as temperature, moisture, and nutritional properties according to different stages^8^.

However, two different methodological perspectives could be applied to the study of the microbiota associated with composting: culture-dependent and culture-independent methods. Owing to their potential to provide complete information on the microbiome during composting, thus avoiding the problems associated with cultivation, molecular techniques have recently become increasingly popular^6^. Next-generation sequencing is considered a practical approach to expanding the repertoire of known biodegrading microorganisms and elucidating their metabolic potential during the different phases of composting.

Several studies have focused on the maturity of compost, but only a handful have been devoted to the alternation between mesophilic and thermophilic microbial communities. As outlined above, the main objective of this study was to establish the relationships between specific microorganisms (bacterial and fungal) and composting phases using next-generation sequencing. Additionally, this study aimed to identify new mesophilic and thermophilic microbial genera throughout the composting of textile waste, which could be beneficial for performing processes that are more efficient and improving final products.

## Results

### Physical–chemical analysis

**Figure 1:**
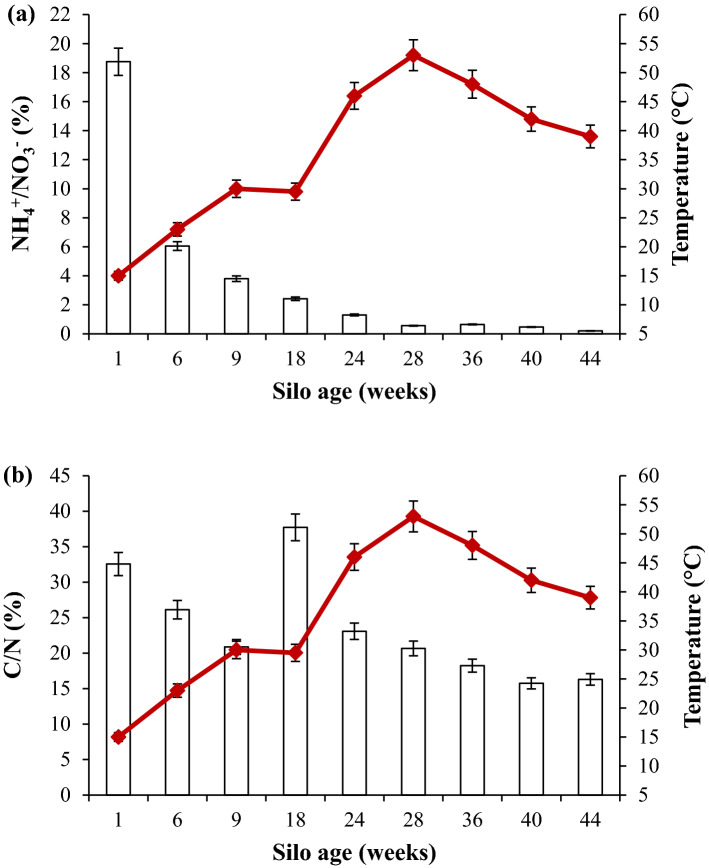
The concentration of NH_4_^+^/NO_3_^−^ ratio (bars) (**a**) and C/N ratio (bars) (**b**) according to temperature (red line) in compost. The values of standard deviation are shown based on three samples.

**Figure 2:**
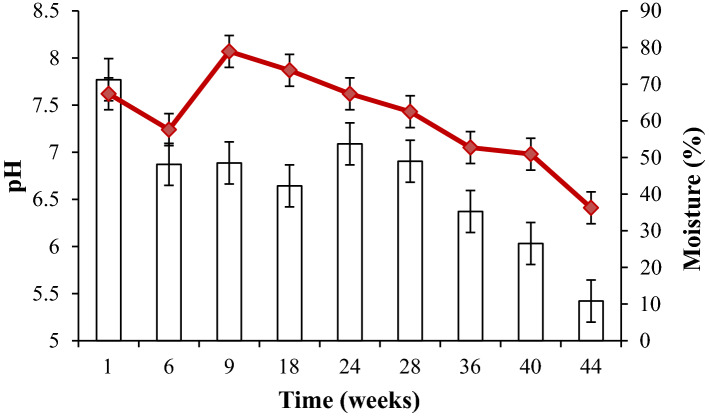
The changes in pH (red line) and moisture (bars) throughout the composting of biomaterial waste^11^. The values of standard deviation are shown based on three samples.

Changes in the C/N and NH_4_^+^/NO_3_^−^ ratios depended on temperature (Fig. 1a,b), and were followed throughout the composting of biomaterial wastes. Figure 1a,b demonstrate that the C/N and NH_4_^+^/NO_3_^−^ ratios decreased from 32.5 to 16.3% and from 18.7 to 0.2%, respectively. The evolution of the C/N and NH_4_^+^/NO_3_^−^ ratios was inversely proportional to the temperature. Indeed, using ANOVA analysis it was demonstrated that temperature had a significant effect on the C/N (*p* = 0.01) and NH_4_^+^/NO_3_^−^ ratios (*p* = 0.0006). According to several authors, a C/N ratio between 15 and 20 and an NH_4_^+^/NO_3_^−^ ratio below 1 reflects a good level of organic matter degradation, thus proving a very advanced degree of maturation of textile waste compost^9,10^. The temperature increased significantly, (*p* = 0.02) from 30 °C on week 9 to 53 °C on week 28, and decreased to 39 °C by the end of composting. Together with the temperature increase, the moisture decreased significantly, (*p* = 0.0004) from 71.2 to 10.8%, and the pH (*p* = 0.0001) decreased from 7.6 to 6.4 by the end of composting (Fig. 2).

### Bacterial diversity of compost samples

Four FASTQ files were generated, corresponding to the pair-end (forward and reverse) sequencing of mesophilic and thermophilic compost samples generated by NGS. Table 1 summarizes the number of 16S rRNA sequences in each phase (mesophilic and thermophilic) of composting using Mothur.

**Table 1:** The evaluation of 16S rRNA sequences using Mothur.

	Composting phase	Starting number of sequences	Number of identified bacterial sequences	Number of actinobacteria (% of identified)
Mix A	Mesophilic phase	124,995	66,280	6.87
	Thermophilic phase	113,551	62,396	9.36

**Figure 3:**
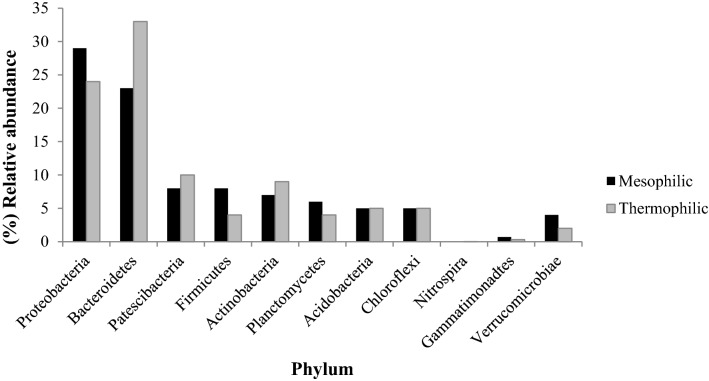
The effect of compost phase on the relative abundance (%) of individual bacterial phyla.

Analysis of Fig. 3 shows that all bacterial phyla were affected at the composting phase. Indeed, it is noteworthy that the bacterial composition of the mesophilic phase is considerably different from that of the thermophilic phase. Bacteroidetes and Proteobacteria were identified as the most dominant phyla detected during both phases of textile waste composting—representing 23% and 29% of total bacteria for the mesophilic phase, and 24% and 33% of total bacteria for the mesophilic phase, respectively (Fig. 3). Patescibacteria, Firmicutes, Actinobacteria, Acidobacteria, Planctomycetes, and Chloroflexi were the other main phyla found in the compost samples. A significant increase in the abundance of Actinobacteria was observed in the thermophilic phase of composting compared to the mesophilic one (Fig. 3). All of these phyla were considerably affected by the composting phase, except for Acidobacteria and Chloroflexi.

In total, 21 genera were detected in the mesophilic and thermophilic phases of textile waste composting. They are presented in Table 2, together with their abundance rates. Sequences associated with *Cellulomonas* (Actinobacteria), *Steroidobacter* (γ-proteobacteria), *Mycobacterium* (Actinobacteria), *Streptomyces* (Actinobacteria), and *Paenibacillus* (Firmicutes) were the most abundant throughout the mesophilic phase (60%, 29%, 23%, 16%, and 13%, respectively). Other genera were also identified during this investigation as organic matter degraders, although in minor abundance—namely *Chitinophaga (*Bacteroidetes), *Nitrosomonas* (Proteobacteria), and *Nitrobacter* (Proteobacteria).

**Table 2:** The contribution of the most abundant bacterial genera during the composting of textile waste.

Phylum	Family	Genus	Contribution to the total number of sequences, %	
			Mesophilic phase	Thermophilic phase
Actinobacteria	Cellulomonadaceae	*Cellulomonas*	0.004	0.00
Proteobacteria	Steroidobacteraceae	*Steroidobacter*	0.12	0.03
Proteobacteria	Xanthomonadaceae	*Pseudoxanthomonas*	0.08	0.13
Proteobacteria	Devosiaceae	*Devosia*	0.25	0.84
Actinobacteria	Mycobacteriaceae	*Mycobacterium*	0.58	0.64
Bacteroidetes	Flavobacteriaceae	*Flavobacterium*	1.28	5.23
Actinobacteria	Streptomycetaceae	*Streptomyces*	0.05	0.02
Firmicutes	Paenibacillaceae	*Paenibacillus*	0.12	0.07
Proteobacteria	Pseudomonadaceae	*Pseudomonas*	0.11	0.40
Proteobacteria	Beijerinckiaceae	*Chelatococcus*	0.009	0.001
Proteobacteria	Alcaligenaceae	*Achromobacter*	0.04	0.09
Firmicutes	Clostridiaceae	*Clostridium*	0.13	0.08
Proteobacteria	Xanthobacteraceae	*Nitrobacter*	0.17	0.15
Actinobacteria	Nocardioidaceae	*Nocardioides*	0.07	0.30
Proteobacteria	Enterobacteriaceae	–	0.20	0.06
Actinobacteria	Micromonosporaceae	*Micromonospora*	0.006	0.14
Bacteroidetes	Bacteroidaceae	*Bacteroides*	0.008	0.00
Actinobacteria	Bifidobacteriaceae	*Bifidobacterium*	0.001	0.00
Proteobacteria	Burkholderiaceae	*Burkholderia*	0.00	0.001
Bacteroidetes	Chitinophagaceae	*Chitinophaga*	0.18	0.003
Proteobacteria	Nitrosomonadaceae	*Nitrosomonas*	0.07	0.05

Notably, there was a decrease in all genera except for *Cellulomonas* in the thermophilic phase, which was entirely absent (Table 2). *Devosia* (α-proteobacteria), *Flavobacterium* (Bacteroidetes), *Pseudoxanthomonas* (Proteobacteria), *Pseudomonas* (γ-proteobacteria), and *Achromobacter* (β-proteobacteria) were the most abundant genera during the thermophilic phase. The abundance of these genera was substantially lower in the mesophilic phase. Additionally, using the UniProt database, the most common enzymatic activities in the mesophilic and thermophilic phases were predicted, thus forecasting the presence of cellulase, hemicellulase, xylanase, pectin depolymerase, and phosphatases (acid and alkaline) in both phases (Table 3).

**Table 3:** The enzymatic profiles detected in bacterial communities involved in organic matter degradation throughout the composting phases (mesophilic and thermophilic), according to the UniProt database.

UniProt entry	Gene	Protein code	Protein type	Mesophilic/thermophilic phase	Bacteria
P10476	*celA*	3.2.1.4	Cellulase	Mesophilic and thermophilic	*Pseudomonas*
P23665	*endA*	3.2.1.4	Cellulase	Mesophilic	*Bacteroides*
P49424	*manA*	3.2.1.78	Hemicellulase (β-Mannanase)	Mesophilic and thermophilic	*Pseudomonas*
P51529	*gmuG,*	3.2.1.78	Hemicellulase (β-Mannanase)	Mesophilic and thermophilic	*Streptomyces*
P07986	*cexX*	3.2.1.8	Xylanase (hemicellulase)	Mesophilic and thermophilic	*Cellulomonas*
P17137	*xynB*	3.2.1.8	Xylanase (hemicellulase)	Mesophilic and thermophilic	*Clostridium*
C6CRVO	*xynA1*	3.2.1.8	Xylanase (hemicellulase)	Mesophilic and thermophilic	*Paenibacillus*
Q59219	*asdII*	3.2.1.55	Hemicellulase	Mesophilic	*Bacteroides*
P94552	*abf2*	3.2.1.55	Hemicellulase	Mesophilic	*Bacillus*
P20041	*pglA*	3.2.1.15	Pectin depolymerase	Mesophilic and thermophilic	*Pseudomonas*
Q05205	*phoA*	3.1.3.1	Alkaline phosphatase	Mesophilic and thermophilic	*Lysobacter*
A1YYW7	*phoK*	3.1.3.1	Alkaline phosphatase	Mesophilic and thermophilic	*Sphingomonas*
O53361	*sapM*	3.1.3.2	Acid phosphatase	Mesophilic and thermophilic	*Mycobacterium*
Q841V6	*abfB*	3.2.1.55	Hemicellulase	Mesophilic	*Bifidobacterium*

### Fungal diversity of compost samples

Four FASTQ files were generated, corresponding to the pair-end (forward and reverse) sequencing of mesophilic and thermophilic compost samples generated by NGS. Table 4 illustrates the number of ITS sequences in both the mesophilic and thermophilic phases of composting.

**Table 4:** The evaluation of ITS sequences using DADA2.

	Composting phase	Starting number of sequences	Number of sequences after filter	Sequences proceed to classification (% of starting amount)
Mix A	Mesophilic phase	177,965	76,525	43.00
	Thermophilic phase	192,732	102,729	53.30

**Figure 4:**
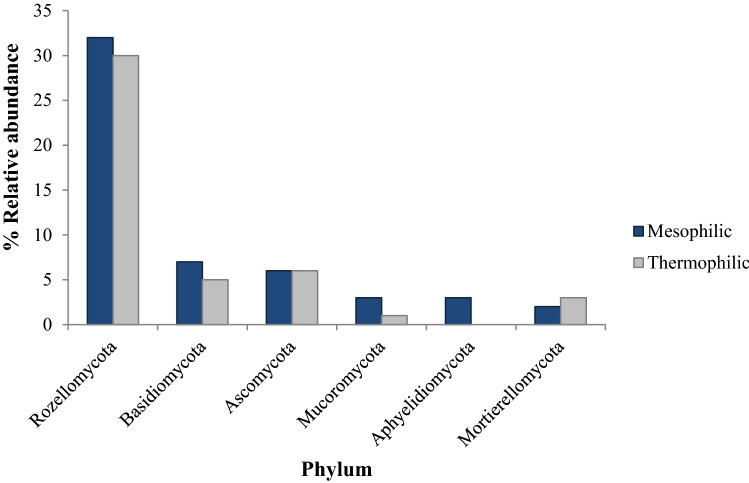
The effect of compost phase on the relative abundance (%) of individual phyla of fungi in compost samples.

The relative abundances of fungi phyla in compost samples were examined at different phases of composting. Figure 4 illustrates that the relative abundance of the members of each phylum was greater in samples from the mesophilic phase than in those from the thermophilic phase. As for the bacteria, their abundance was also dependent on the composting phase. Indeed, the most abundant phyla detected in both phases of composting were Rozellomycota, Basidiomycota, and Ascomycota—amounting to 32%, 7%, and 6% for the mesophilic phase, and 30%, 5%, and 6% for the thermophilic phase, respectively. Mucoromycota, Aphyelidiomycota, and Mortierellomycota were observed in lower abundances (Fig. 4). All of the phyla detected were affected by the composting phase, with the exception of Ascomycota; there was no significant difference in abundance in either phase.

At the class level, Sordariomycetes, Tremellomycetes, Agaricomycetes, Eurotiomycetes, Saccharomycetes, Orbiliomycetes, Pezizomycetes, and Dothideomyctes were present in both phases. Indeed, Sordariomycetes, Tremellomycetes, Saccharomycetes, Orbiliomycetes, and Dothideomyctes (57%, 44%, 7%, 6%, and 3%, respectively) were the most abundant fungal classes in the mesophilic phase. In contrast, Agaricomycetes, Eurotiomycetes, and Pezizomycetes (35%, 23%, and 10%, respectively) prevailed in the thermophilic phase.

Table 5 illustrates a comparison between the most abundant genera identified in both phases. Samples from either phase of composting showed several similarities, with differences in abundance. Some taxa were specific to the mesophilic phase, others to the thermophilic. In this regard, *Fusarium*, *Acremonium*, *Penicillium*, and *Rhizopus* were detected as the most frequently identified genera during composting, but with a generally higher abundance in the thermophilic phase compared to the mesophilic. In the mesophilic phase of composting, *Aspergillus* and *Cyphellophora* were present, while for the thermophilic phase, *Fusarium*, *Acremonium*, *Penicillium*, *Rhizopus*, and *Phialophora* were the most abundant genera. Still, some exceptions were noted with some genera from the list of abundant fungi present in the mesophilic phase that were not present in the thermophilic phase, and vice-versa (Table 5).

**Table 5:** The contribution of the most abundant fungi genera during the composting of textile waste.

Phylum	Family	Genus	Presence, %	
			Mesophilic phase	Thermophilic phase
Ascomycota	Nectriaceae	*Fusarium*	1.26	1.33
Ascomycota	Hypocreaceae	*Acremonium*	0.63	1.27
Ascomycota	Aspergillaceae	*Penicillium*	0.27	0.67
Ascomycota	Aspergillaceae	*Aspergillus*	0.09	0.01
Zygomycota	Rhizopodaceae	*Rhizopus*	2.91	0.96
Ascomycota	Cyphellophoraceae	*Cyphellophora*	0.15	0.00
Basidiomycota	Trichosporonaceae	*Apiotrichum*	2.72	1.50
Mortierellomycota	Mortierellaceae	*Mortierella*	2.45	3.30
Ascomycota	Herpotrichiellaceae	*Phialophora*	0.07	0.62

Using the UniProt database, fungal cellulase, hemicellulase, xylanase, pectin depolymerase, and phosphatase activities were predicted to be present in both phases (Table 6).

**Table 6:** The enzymatic profiles detected in fungi communities involved in organic matter degradation throughout the composting phases (mesophilic and thermophilic), according to the UniProt database.

UniProt entry	Gene	Protein code	Protein type	Mesophilic/thermophilic phase	Fungi
Q12679	*cekA*	3.2.1.4	Cellulase	Mesophilic and thermophilic	*Aspergillus*
Q5B7X2	*manA*	3.2.1.78	Hemicellulase (β-Mannanase)	Mesophilic and thermophilic	*Aspergillus*
W0HJ53	*xyn1*	3.2.1.8	Xylanase (hemicellulase)	Mesophilic and thermophilic	*Rhizopus*
Q9P8J1	*xynA*	3.2.1.8	Xylanase (hemicellulase)	Mesophilic and thermophilic	*Penicillium*
I1S3T9	*xynC*	3.2.1.8	Xylanase (hemicellulase)	Mesophilic and thermophilic	*Fusarium*
Q12549	*xyn5*	3.2.1.8	Xylanase (hemicellulase)	Mesophilic and thermophilic	*Aspergillus*
O59925	*pepg1*	3.2.1.15	Pectin depolymerase	Mesophilic and thermophilic	*Penicillium*
P37274	*phoA*	3.1.3.2	Acid phosphatase	Mesophilic and thermophilic	*Penicillium*
P20584	*pacA*	3.1.3.2	Acid phosphatase	Mesophilic and thermophilic	*Aspergillus*

## Discussion

The biodegradation of organic matter by microorganisms is the primary component of the transformation of organic waste during composting. In this study, NGS analysis indicated that the mixture under investigation harbored many different microbial genera, thus proving that compost formed from textile waste is an ideal habitat for various new mesophilic and thermophilic microbes. Our findings are supported by those previously obtained, which demonstrated an increase in the concentration of microorganisms (bacteria, actinomycetes, and fungi) during the composting of textile waste. They also reflect the level of diversity of the microbial community in the feedstock and the high degree of functional redundancy known to exist in these microbial communities. In this regard, different metabolic pathways lead to significant changes in the physical–chemical parameters of the substrate, which in turn lead to community changes and influence microbial abundance and the succession of microorganisms. Several phases occur during the composting of textile waste. At the beginning of composting, temperatures below 45 °C characterize the mesophilic phase (Fig. 1). During the mesophilic phase, the availability of favorable conditions (in terms of nutrition, temperature, moisture, etc.) stimulates the proliferation of mesophilic microorganisms, thus activating their metabolism of easily metabolizable substrates—such as carbohydrates and free amino acids. The rise in temperature and the decrease in moisture observed are mainly due to the evaporation of water. Such temperatures (up to 50 °C or even 75 °C) induce the appearance of thermotolerant and thermophilic microorganisms, characterizing the thermophilic phase. During this phase, a significant proportion of the organic matter is transformed by thermophilic microorganisms mineralizing organic carbon and releasing CO_2_ and nitrogen compounds, thus decreasing the C/N ratio^10,12^. A decrease in the C/N ratio to a value below 20 was recorded, thus demonstrating a rise in the humification rate and the degradation of recalcitrant molecules such as lignin, hemicelluloses, and cellulose—a good indicator of compost maturity. At the same time, a decrease in the NH_4_^+^/NO_3_^−^ ratio to a value below 1 was observed, which is a good indicator of compost maturity. This decrease could be attributed to the phenomenon of nitrification and/or the denitrification of inorganic nitrogen. A remarkable initial increase in pH followed by a swift decrease was also observed. The rise in pH could be assigned to the ammonification carried out by microorganisms. In contrast, the subsequent decrease in pH could be attributed to the degradation of organic acids by the microorganisms identified^11^. Although composting is an ancient treatment, it is a very complex process as a result of the numerous changes in physical–chemical and biological states that occur over its course^11^. A good understanding of these changes requires careful study of the succession of microbial communities that comprise all of the microorganisms present, including those in tiny proportions^13^. Such an investigation could establish a microorganism database for each of the composting phases according to physical–chemical parameters^14^. The use of the NGS approach allows the achievement of this goal. During such an analysis, a huge amount of bacterial, actinomycete, and fungal species were identified. In this respect, bacteria are considered among the most dominant microorganisms in quantity and diversity during composting^15,16^. The authors’ previous work has demonstrated that bacterial concentration in compost samples is higher than fungal and actinomycete concentrations during textile waste composting^11^. Such an abundance is because of their small size and their ability to proliferate at a wide range of pH and temperature intervals^17^. The dominance of the genera belonging to Proteobacteria, Bacteroidetes, Actinobacteria, and Firmicutes phyla is expected; it is in agreement with previous results^1,13^. This demonstrates that, during composting, the most abundant bacteria are those belonging to the phyla mentioned above, owing to their ubiquitous nature^2,7,18,19^. Meanwhile, many genera were identified in this study as degrading recalcitrant molecules (lignocellulosic compounds) through their enzymatic system: *Devosia, Flavobacterium*, *Pseudoxanthomonas*, *Pseudomonas*, and *Achromobacter*^11,18,20^*.* Aside from this, the *Devosia* genus was described as a degrader of organic sulfur, phosphorus, and aromatic compounds according to Chandni et al.^21^. Several authors have demonstrated that *Paenibacillus* (belonging to the Firmicutes) could utilize different enzymatic activities to destroy the lignocellulosic compounds such as cellulose, xylose, and hemicelluloses^17,22^. This is highlighted using the UniProt database (Table 3) and via the analysis of enzymatic activities in a previous study by the authors of this paper, thus revealing a huge concentration of different enzymatic activities during the composting of textile waste^11^. Such genera could explain the rise in temperature due to active metabolism and, therefore, the decaying organic matter represented by the decrease in the C/N ratio.

Other genera were also identified during this investigation as organic matter degraders, although in minor abundance—namely *Chitinophaga*, *Nitrosomonas*, and *Nitrobacter*. *Chitinophaga* (belonging to Bacteroidetes) was recently identified as cellulose-degrading species during the mesophilic phase, and was found to be able to grow on solubilized forms of cellulose from green-waste compost^2^. Interestingly, three genera associated with the nitrification phenomenon—namely *Steroidobacter*, *Nitrosomonas*, and *Nitrobacter*—were identified. The numbers of nitrate- (*Nitrobacter*) and ammonium-oxidizing bacteria (*Nitrosomonas*) rose markedly through the composting processes. *Steroidobacter* is capable of denitrification using only a narrow range of organic substrates and nitrate as the electron acceptor^23^. All of these genera are nitrogen transformers, and can degrade organic matter into products capable of supporting plant growth by providing the necessary nitrogen. Our findings could be used to explain the changes in the NH_4_^+^/NO_3_^−^ ratio observed during this investigation. Ultimately, using the rarefaction curves (Supplementary Fig. S1), we can state that the NGS performed during the current investigation can describe the majority of bacteria present in the samples analyzed.

Additionally, actinomycetes are multicellular filamentous bacteria acting at the later stages of composting. An increase in the concentration of actinomycetes during the thermophilic phase has previously been reported^17^. This observation is corroborated in the current study, as a significant increase in actinomycete abundance was recorded during the thermophilic phase. This strengthens previous results, demonstrating that the concentration of actinomycetes in compost samples reached peak values during the thermophilic phase^11^. Actinomycetes are essential agents that act on lignocellulose during the thermophilic phase, although their ability to degrade cellulose and lignin is not as extensive as that of fungi. Still, they can degrade cellulose, hemicellulose, and lignin^10,19,24,25^. This finding is consistent with the results obtained during this investigation, which show that genera from the Actinobacteria phyla (*Streptomyces* and *Mycobacterium*, etc.) are abundant in the thermophilic stages of composting, and have demonstrated their ability to metabolize complex organic compounds—except in the case of *Cellulomonas,* which is not conspicuous in the thermophilic phase. This could be assigned to the fact that bacteria of this genus cannot survive at high temperatures. The enzymatic activities (especially of the xylanase) of *Cellulomonas* were more remarkable in the mesophilic phase (37 °C).

All of the genera detected during both phases possess several enzymes (Table 3) involved directly in the degradation of organic matter—such as cellulase, hemicellulase (β-mannanase), pectin depolymerase, and xylanase (hemicellulase), which strengthens the above explanation. Additionally, several cellulolytic, amylolytic, and proteolytic bacteria appeared in greater concentrations in the thermophilic phase than in the mesophilic phase^18^. The high concentration of cellulolytic microorganisms during the thermophilic phase could be explained by high temperatures that favor the degradation of cellulose^26^, thus proving the major role played by thermophilic microorganisms in decaying cellulose during the composting of textile waste. Notably, sequences associated with *Escherichia*, *Salmonella*, or *Listeria* were not detected, which demonstrates that composting is an effective process for the destruction of pathogens and pathogen indicators owing to multiple heating cycles during composting^18^.

Interestingly, fungi can decay the organic matter not transformed by bacteria. Several authors found that Sordariomycetes and Eurotiomycetes are the most abundant eukaryotes in both phases of the composting process^8,27^. Variations in fungal communities corresponding to different phases (mesophilic and thermophilic) could be explained by changes in the physical–chemical characteristics of the compost highlighted during this investigation, such as pH, temperature, and moisture content. Temperature is the most critical factor affecting fungal growth. Most fungi are mesophilic, and thrive between 5 and 37 °C, with an optimum temperature of 25–30 °C^28,29^, but some also grow at higher temperatures. For example, *Penicillium* and *Fusarium*, which have been the subject of numerous studies, have demonstrated the ability to produce enzymes active in cellulose and lignin degradation even at high temperatures of 46–49 °C^8^. The presence of *Penicillium*, *Aspergillus*, *Acremonium*, and *Fusarium* in compost samples was expected. *Penicillium* is ubiquitous in the environment, and plays a vital role in decaying organic matter (lignin, cellulose, or hemicelluloses)^30,31^. *Aspergillus*, *Acremonium*, and *Fusarium* are associated with the degradation of organic matter (lignocellulosic compounds, cellulose, and hemicellulose). These findings are supported by the prediction obtained from the UniProt database (Table 6) and from previous studies^11^, thus demonstrating the ability of these genera to produce a wide range of enzymatic activities. This allows fungi to degrade organic matter even at high temperatures (in the thermophilic phase), and corroborates the results of the current study regarding the C/N and NH_4_^+^/NO_3_^−^ ratios.

Indeed, the composting process generates high temperatures, demonstrating the crucial role of small groups of thermophilic fungi in the biodegradation of organic matter. Generally, fungi are not detected in the thermophilic phase of composting^24,26,29^. Remarkably, the added value of this study lies in the presence of a high abundance of thermophilic fungi in this phase (Table 5), suggesting that thermophilic fungi have a significant role in the degradation of organic matter during the thermophilic phase of textile waste composting. This finding could be applied when selecting new thermophilic fungi and reusing them to improve the composting performance of textile waste and produce mature compost. Eventually, analysing the rarefaction curves for fungi, it can be concluded that the description of the fungal diversity provided by NGS was effective (Supplementary Fig. S2).

This investigation depicted a detailed study of the textile waste composting process from a molecular microbiology standpoint. Using the next-generation sequencing approach, the microbiota variation that orchestrates the composting process and the change of enzymatic activities was described. These findings allow for the generation of a list of bacteria and fungi and their enzymes that are responsible for decaying lignocellulosic material. The impressive variety of bacterial and fungal microorganisms and metabolic functions during both the mesophilic and thermophilic phases of composting warrant the results obtained during the composting of the textile waste, and prove that the resulting compost meets the required level of maturity. Ultimately, it can be concluded that compost from textile waste is a treasure trove of new microorganisms and enzymes that are beneficial for the degradation of organic matter. Interestingly, this investigation could be used as a database for the dominant microorganisms involved in the degradation of organic matter. This would allow for the selection of new bacterial and thermophilic fungi genera and their reuse in bioaugmentation to improve the performance and reduce the duration of the composting of textile waste, and to produce mature compost.

## Methods

### Experimental design and setup

The preparation of the mixture was performed according to a previously established procedure^5^. The composting experiment was carried out using an in-silo composter of approximately 200 L (with an effective size of 0.58 × 0.58 × 0.92 m). A mixture was established for composting shredded waste, labelled ‘Mix A’ (with a 40%/30%/30% ratio of textile/green/paper and cardboard waste). After the appropriate components were mixed, the silo was turned at least three times per week for 44 weeks. Samples were collected according to the four cardinal positions (north, east, south, and west) from three depths (0–20 cm, 30–40 cm, and 50–70 cm) in triplicate each week (in weeks 1, 6, 9, 18, 24, 28, 36, 40, and 44), and were then placed into polythene bags and stored at 4 °C until further analysis was conducted.

### Experimental analysis

A calibrated pH meter was used to measure pH (Hanna Hl-10530 pH meter). Moisture was analyzed according to the protocol described by the French Association for Standardization—AFNOR^32^. The C/N ratio for the initial mix and the end products was calculated by analyzing the total C and N percentages using a TOC analyzer (Shimadzu-V CSN)^33^. The total ammonium and nitrate ion (NH_4_^+^/NO_3_^−^) ratios for the pre-composted mix and the final products were calculated from the percentage of NH_4_^+^ and NO_3_^−^. This was performed using the method based on alkaline distillation for ammonium ions (NH_4_^+^), and using reduction by Dewarda alloying for nitrate ions (NO_3_^−^)^9^.

### Temperature

Temperature was measured in triplicate each week. The silo was divided into three depths. After turning, measurements were taken from each of these three areas to allow for the equilibration of silo temperature after the incorporation of cooler air by inversion. The temperature was monitored using an all-sun ETP109B Digital Thermometer. To measure the inner temperature, the probe was pushed to the maximum depth of the silo, while the outer temperature was measured close to the surface. Both readings were then averaged.

### DNA extraction

DNA was extracted from compost samples which were taken from the silo during both the mesophilic (week 9) and thermophilic phases (week 28) of the process. This was done using the PureLink Microbiome DNA Purification Kit following the manufacturers’ instructions, and was stored at − 20 °C until use. The quantity and quality of the extracted DNA were initially checked using agarose gel electrophoresis, and the samples later passed quality control at Macrogen before amplification and sequencing.

### Next-generation sequencing of 16S rRNA and ITS gene amplicons

The sequencing and amplification of 16S rRNA or Internal Transcribed Spacer (ITS) was performed at the Genomic Analysis Platform Macrogen (Republic of Korea), using the Illumina sequencing platform.

The following oligonucleotide sequences were used for the amplification of the 16S rRNA gene, targeting the V3–V4 region from 16S rRNA primers specific to bacteria:

Forward:

5′-TCGTCGGCAGCGTCAGATGTGTATAAGAGACAGCCTACGGGNGGCWGCAG.

Reverse:

5′-GTCTCGTGGGCTCGGAGATGTGTATAAGAGACAGGACTACHVGGGTATCTAATCC.

For the fungi, the internal transcribed ITS2 spacer region was amplified using forward primer ITS3 (5′-GCATCGATGAAGAACGCAGC-3′) and reverse primer ITS4 (5′-TCCTCCGCTTATTGATATGC-3′).

### Processing and analysis of the sequencing data

Bioinformatics analysis and data annotation were performed by using Mothur to analyze 16S rRNA gene fragments for bacteria and DADA2 to analyze ITS regions for fungi. After demultiplexing the raw FASTQ files using Mothur v 1.44.3^34^, the files generated from the paired-end sequencing were filtered and aligned with the SILVA v132 database. Fungal sequences were then trimmed using CUTADAPT to remove primer sequences and, finally, sorted according to barcode. The selected region of fungal origin was then filtered using DADA2, an open-source pipeline that uses a different algorithm for sequence clustering, which was then aligned with the UNITE 8.0 database^35^. Rarefaction curves were created to see the bacterial and fungal diversity of the samples; these curves were added in the supplementary materials. Enzymatic activities were predicted using the UniProt (Universal Protein) database, a comprehensive resource for protein sequence and annotation data that allows different protein sequences to be searched for, coding for a wide range of enzymatic proteins. In this way, bacterial and fungal genera found in samples were searched for in the UniProt database. The proteins encoding genes were also searched for in sequenced species from the genera found. All of these results were analyzed using ANOVA (normalized analysis of variance), carried out using the GraphPad Prism.

## Supplementary Information

Supplementary Figures.
