# Supplementary material for: Microbial community dynamics in the mesophilic and thermophilic phases of textile waste composting identified through next-generation sequencing
Source: Sci Rep. 2021 Dec 8;11:23624. doi: 10.1038/s41598-021-03191-1 (PMC8654937; doi:10.1038/s41598-021-03191-1)
Supplement: Supplementary file 1 — Supplementary Figures. [file 41598_2021_3191_MOESM1_ESM.docx]

**Supplement Material**


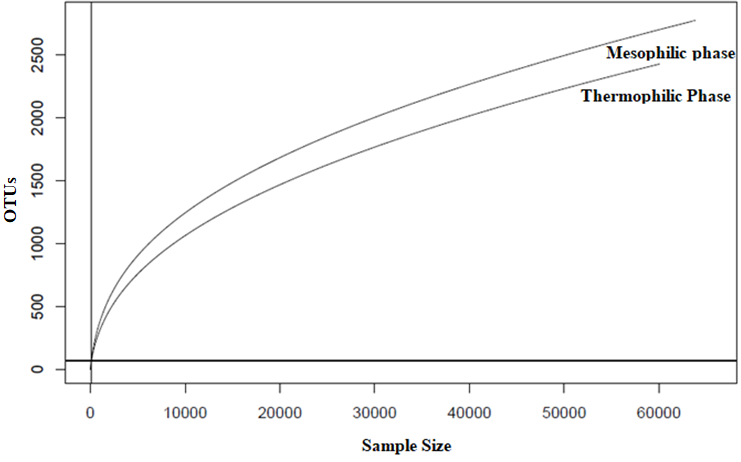


**Figure S1.** Rarefaction curves for bacterial diversity (OTUs).

**
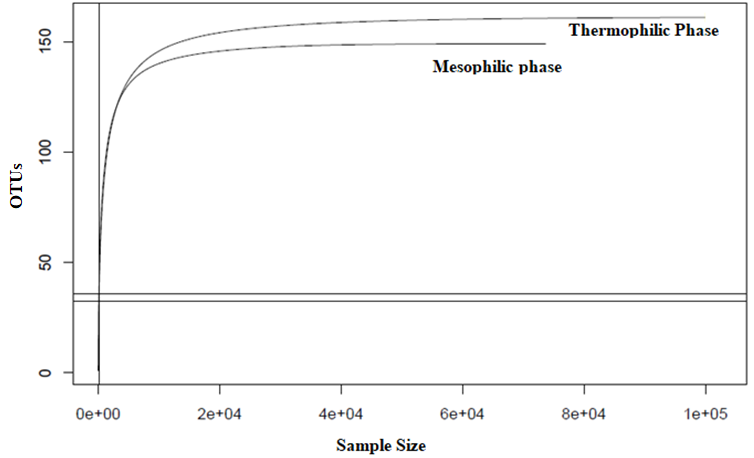
**

**Figure S2.** Rarefaction curves for fungal diversity (OTUs).
